# High expression of SMYD3 indicates poor survival outcome and promotes tumour progression through an IGF-1R/AKT/E2F-1 positive feedback loop in bladder cancer

**DOI:** 10.18632/aging.102718

**Published:** 2020-02-01

**Authors:** Guoliang Wang, Yi Huang, Feilong Yang, Xiaojun Tian, Kun Wang, Li Liu, Yidong Fan, Xiaofeng Li, Luchao Li, Benkang Shi, Yichang Hao, Chuanyou Xia, Qingsheng Nie, Yue Xin, Zhenfeng Shi, Lulin Ma, Dawei Xu, Cheng Liu

**Affiliations:** 1 Department of Urology, Peking University Third Hospital, Beijing, China; 2 Department of Urology, Tianjin Medical University Cancer Institute and Hospital, National Clinical Research Centre for Cancer, Key Lab for Cancer Prevention and Therapy, Tianjin, China; 3 School of Nursing, Beijing University of Chinese Medicine, Beijing, China; 4 Department of Urology, Shandong University Qilu Hospital, Jinan, China; 5 Department of Medicine, Bioclinicum and Centre for Molecular Medicine, Karolinska University Hospital Solna and Karolinska Institutet, Stockholm, Sweden; 6 Department of Urology, The Central Hospital of Zibo, Zibo, China; 7 Department of Urology, Chifeng University Second Hospital, Chifeng, China; 8 Department of Urology, The People’s Hospital of Xinjiang Uyghur Autonomous Region, Xinjiang, China; 9 Karolinska Institute-Shandong University Collaborative Laboratory for Cancer and Stem Cell Research, Jinan, China

**Keywords:** SMYD3, bladder cancer, AKT, IGF1R, E2F-1

## Abstract

The AKT/mTOR pathway is critical for bladder cancer (BC) pathogenesis and is hyper-activated during BC progression. In the present study, we identified a novel positive feedback loop involving oncogenic factors histone methyltransferase SMYD3, insulin-like growth factor-1 receptor (IGF-1R), AKT, and E2F-1. SMYD3 expression was significantly up-regulated in BC tumors and positively associated with histological grade, lymph node metastasis, and shorter patient survival. Depletion of SMYD3 inhibited BC cell proliferation, colony formation, migration, invasion, and xenograft tumor growth. Mechanistically, SMYD3 inhibition led to the diminished AKT/mTOR signaling activity, thereby triggering deleterious effects on BC cells. Furthermore, SMYD3 directly activates the expression of IGF-1R, a critical activator of AKT in BC, by inducing hyper-methylation of histone H3-K4 and subsequent chromatin remodeling in the IGF-1R promoter region. On the other hand, E2F-1, a downstream factor of the AKT pathway, binds to the E2F-1 binding motifs at the SMYD3 promoter and consequently induces SMYD3 transcription and expression. Thus, SMYD3/IGF-1R/AKT/E2F-1 forms a positive feedback loop leading to the hyper-activated AKT signaling. Our findings provide not only profound insights into SMYD3-mediated oncogenic activity but also present a unique avenue for treating BC by directly disrupting this signaling circuit.

## INTRODUCTION

Bladder cancer (BC) is the most common type of urological malignancies, and more than 90% of them are urothelial carcinomas [1]. The driving factors underlying the BC pathogenesis remain incompletely defined. Its initiation and progression have been recognized as a complex process that involves both genetic and epigenetic alterations [2, 3]. Evidence has indicated the importance of aberrant cellular signaling in BC. For instance, the hyper-activated AKT/mTOR signaling cascade as a key driver is observed in up to 40% of BC [4–6]. Therefore several types of small molecule inhibitors targeting the AKT/mTOR pathway in BC is under development [7]. It has been well established that insulin-like growth factor-1 receptor (IGF-1R), an activator of AKT/mTOR, is overexpressed in BC and serves as a useful prognostic factor [8–10]. However, the mechanism underlying the IGF-1R/ AKT/mTOR axis activation in BC is still elusive.

SET and MYND domain-containing protein 3 (SMYD3) is a histone methyltransferase targeting histone H3-K4 for its di/trimethylation [11]. Moreover, SMYD3 directly recognizes and occupies its target promoters by binding to the motif/s (5′-CCCTCC-3′), and methylates H3-K4, thereby leading to chromatin remodeling and transcriptional activation [12]. Intriguingly, a recent study showed that methylation of a non-histone protein MAP3K2 by SMYD3 increased MAP kinase signaling and promotes oncogenesis, which suggests that SMYD3 has much broader roles in carcinogenesis [13]. In general, SMYD3 expression is very weak or undetectable in the majority of normal human tissues, whereas its overexpression has been implicated in the development and progression of colorectal, hepatocellular and prostate cancer [14, 15]. A recent report showed that SMYD3 was also over-expressed in BC and facilitated BC progression [16], however, it is currently unclear how SMYD3 accomplishes its ends in BC, which is the key issue addressed in the present study. We observed that SMYD3 expression was aberrantly induced in BC tumors and predicted a poor patient outcome. The overexpressed SMYD3 transcriptionally up-regulated the expression of IGF-1R, through which the AKT/mTOR pathway was activated in BC cells, while the AKT downstream effector E2F1 further enhanced *SMYD3* transcription. Thus, SMYD3 serves as a bridge to form a positive feedback loop with IGF-1R, Akt, and E2F-1, thereby amplifying the AKT signaling and promoting BC pathogenesis.

## RESULTS

### SMYD3 expression is upregulated in primary BC tumors and predicts poor patient outcomes

**Figure 1:**
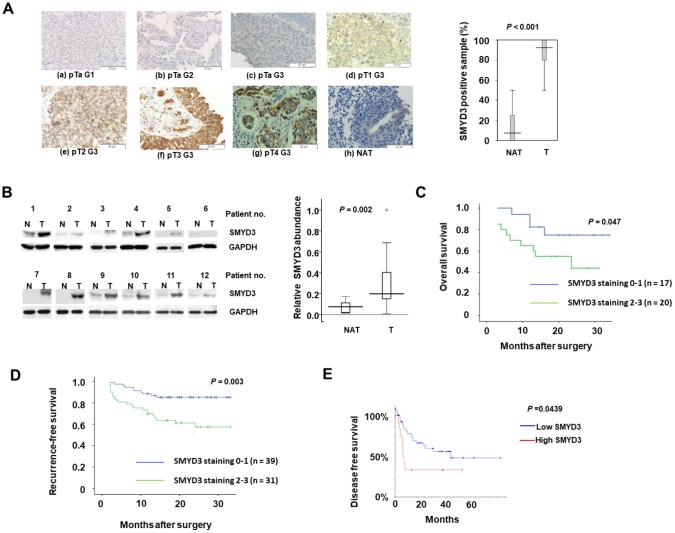
**SMYD3 is upregulated in BC tumors and serves as a poor prognostic indicator of BC.** (**A**) SMYD3 expression in representative BC tumors (T) and NAT (please define NAT: non-cancerous adjacent tissues?) (n=65) analyzed using IHC (Magnification: ×200). The Tukey box-and whiskers plot shows the percentage of positive IHC staining SMYD3 in 65 BC tumors and NAT. A set of Wilcoxin tests for paired groups was used to calculate the two-sided P values. (a-g: BC tumors; h: NAT of f). (**B**) Representative images of Western blot analysis of SMYD3 expression in 25 pairs of frozen BC tumors (T) and corresponding non-cancerous bladder tissues (N). The Tukey box-and whiskers plot shows the quantification of the Western blot signals. A set of Wilcoxin tests for paired samples was used to calculate the two-sided P values. (**C**) Kaplan-Meier plot comparing survival of BC patients undertaken radical cystectomy based upon SMYD3 expression. Statistical significance was determined by log-rank test. (**D**) Kaplan-Meier plot comparing survival of recurrence of BC patients undertaken TURBT (Define it) based upon SMYD3 expression. Statistical significance was determined by log-rank test. (**E**) Kaplan-Meier plot comparing DFS of BC patients according SMYD3 mRNA abundance from the TCGA dataset and analyses were described in Materials and Methods. Statistical significance was determined by log-rank test.

We first determined SMYD3 protein expression in primary tumors from 65 BC patients using IHC. Fifty-eight out of 65 (89.2%) cases had SMYD3 expression in their BC tumors, while only 5 out of 65 (7.7%) of the matched normal tissues exhibited weak positive cytoplasmic staining (*P*<0.001, *χ*^2^-test) (Figure 1A). The level of SMYD3 expression was positively correlated with tumor stage and lymph node metastasis (Table 1). Moreover, significantly higher levels of SMYD3 expression were observed in G3 tumors compared to those in G2 and G1 (*P* = 0.029, *χ*^2^-test), while there was no significant difference between G1 and G2 (*P* = 0.530, *χ*^2^-test) (Table 1). In addition, we did not observe a significant difference in SMYD3 expression between muscle invasive (≥pT2) and non-invasive BC (<pT2) (*P* = 0.446, *χ*^2^-test). To further verify IHC results, we performed a Western blot analysis on 25 freshly frozen BCs and their matched normal counterparts from the above patients. Enhanced SMYD3 expression was observed in 23 of 25 tumors compared with their corresponding non-cancerous tissues (Figure 1B). These data clearly demonstrated SMYD3 over-expression in the majority of primary BCs.

**Table 1:** SMYD3 expression in relation to clinicopathologic variables in 65 BC patients undertaken cystectomy.

**Variable**	**Total**	**SMYD3 staining (%)**		***P*-value**
		**0–1**	**2–3**	
Sex				
Male	53 (81.5)	38 (58.5)	15 (15.4)	0.642
Female	12 (18.5)	10 (15.4)	2 (3.1)	
Age, years (median 64)				
< 64	30 (46.2)	22 (33.8)	8 (12.3)	0.931
≥64	35 (53.8)	26 (40.0)	9 (13.9)	
Lymph node metastasis				
No	59 (90.8)	46 (70.8)	13 (20.0)	0.036
Yes	6 (9.2)	2 (3.1)	4 (6.2)	
Histological grade				
G1	9 (13.9)	9 (13.9)	0 (0.0)	
G2	19 (29.2)	16 (24.6)	3 (4.6)	0.029
G3	37 (56.9)	23 (35.4)	14 (21.5)	(G1/2vsG3)
Muscle invasive				
No	18 (27.7)	15 (23.1)	3 (4.6)	0. 446
Yes	47 (72.3)	33 (50.8)	14 (21.5)	

0–1: low-SMYD3 expression; 2–3: high-SMYD3expression. Associations between SMYD3 immunostaining level and clinicopathological parameters were analyzed with *χ*^2^-test.

Survival analysis revealed that SMYD3 up-regulation was significantly associated with shorter survival in BC patients undertaken radical cystectomy (Figure 1C). In addition, we examined the SMYD3 expression in 70 BC tissues obtained in transurethral resection of bladder tumors (TURBT) (Supplementary Table 2). Consistently, SMYD3 expression was inversely correlated with recurrence-free survival after TURBT (Figure 1D). The analysis of TCGA database similarly showed that the BC patient group with higher SMYD3 mRNA expression had a significantly shorter PFS (Figure 1E). These data suggested that SMYD3 is a prognostic factor for poor outcomes of BC.

### SMYD3 depletion impairs the tumorigenic potential of BC-derived T24 and 5637 cells

**Figure 2:**
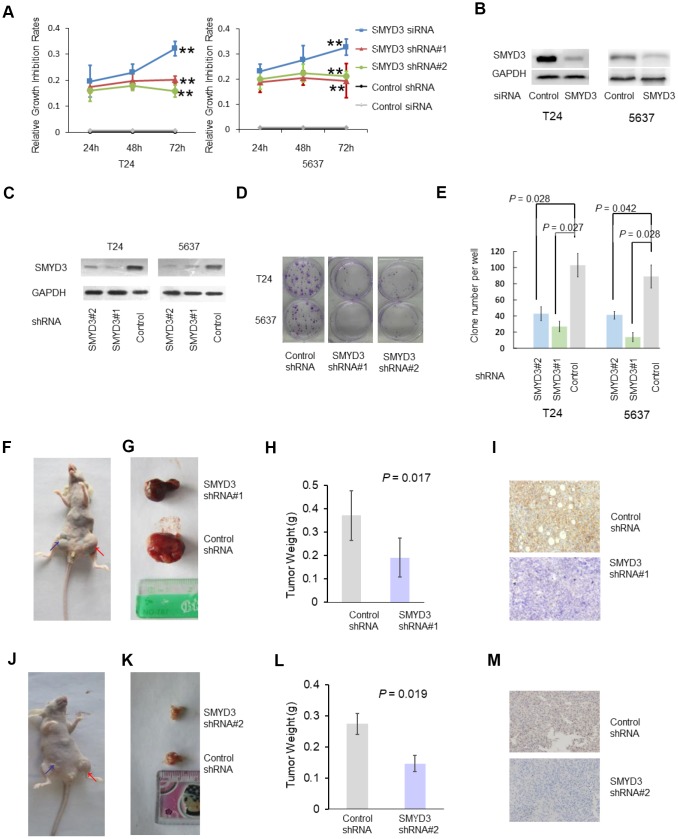
**SMYD3 is required for tumorigenecity of T24 and 5637 cell lines.** (**A**) A CCK-8 assay was performed to measure the growth rate of T24 and 5637 cells at 24, 48 and 72 h post SMYD3 siRNA treatment or shRNA vector transfection. Three independent transfections were performed in triplicate. Two-way ANOVA test were used to calculate the two-sided *P* values. ** *P* < 0.01. (**B**) Western blot analysis of SMYD3 protein expression in T24 and 5637 cells transfected with SMYD3 siRNA for 72 h (n=3). (**C**) Western blot analysis of SMYD3 expression in BC cells stably transfected with the SMYD3 shRNA vector #1, #2 or control vector. GAPDH served as a loading control (n=3). (**D**) Representative images of clonogenic assays of the T24 and 5637 cell lines stably expressing SMYD3 shRNA #1 and #2 or control shRNA. Briefly, 200 cells/well (in 6-well plates) were incubated for 14 days (n=6). (**E**) Quantification of clonogenic assays for 6 independent transfections. Wilcoxon signed-rank tests for paired samples were used to calculate the two-sided *P* values. (**F**–**M**) Xenograft model of BC in nude mice. T24 (**F**–**I**) and 5637 (**J**–**M**) Cells stably expressing SMYD3 shRNA or control shRNA were injected subcutaneously into BALB/c nude mice in the inguinal area (n = 8), and tumor sizes, weights and morphology were evaluated 6 weeks after injection. (**F**, **J**) Representative nude mice injected with BC cells expressing SMYD3-shRNA (blue arrow) or Control shRNA (red arrow). (**G**, **K**) Representative tumors derived from BC cell-injected nude mice. (**H**, **L**) Tumor weights of BC cells expressing SMYD3-shRNA or con-shRNA (*t*-test). (**I**, **M**) IHC of tumor sections from cell-injected nude-mice using SMYD3 antibody; representative staining is shown. Bars: standard deviations (SD).

Given the widespread increase in SMYD3 expression in BC tissues and its association with poor patient outcomes, we sought to explore the biological and functional significance of SMYD3 in BC using cell lines T24 and 5637. The proliferation of T24 and 5637 cells transfected with SMYD3 siRNA was monitored using a CCK8 assay. SMYD3 depletion by siRNA or shRNA led to significant inhibition of cell proliferation (Figure 2A). The efficient silencing of SMYD3 expression was verified by Western blotting (Figure 2B and 2C). To determine whether SMYD3 is required for colony formation, BC cells stably expressing SMYD3 shRNA were established (Figure 2C). T24 and 5637 cells stably expressing SMYD3 shRNA produced a significantly lower number and smaller sized colonies than the same cells transfected with control shRNA *in vitro* (T24-Con-shRNA: 104.7, T24-SMYD3-shRNA#1: 28.0, T24-SMYD3-shRNA#2: 42.3 per well; 5637-Con-shRNA: 85.3; 5637-SMYD3-shRNA#1: 19.0, 5637-SMYD3-shRNA#2: 43.8 per well) (Figure 2D, 2E). We next performed tumor formation experiments with a xenograft model of BC in nude mice using BC cells expressing T24-SMYD3-shRNA#1, 5637-SMYD3-shRNA#2 or Con-shRNA. Nude mice were inoculated subcutaneously in the inguinal area at 0.8 × 10^6^ cells per injection site and sacrificed for evaluation six weeks post-xenotransplantation. Consistent with the *in vitro* data, significantly smaller tumors were observed in mice receiving T24 and 5637 cells expressing SMYD3 shRNA (T24-SMYD3-shRNA#1 vs T24-Con-shRNA = 0.191 vs 0.371; 5637-SMYD3-shRNA#2 vs 5637-Con-shRNA = 0.146 vs 0.274) (Figure 2F–2M). Thus, SMYD3 depletion significantly suppressed tumor growth and oncogenic potential of BC cells both *in vitro* and *in vivo*.

### SMYD3 depletion induces apoptosis and slows down cell cycle progression of BC cells

**Figure 3:**
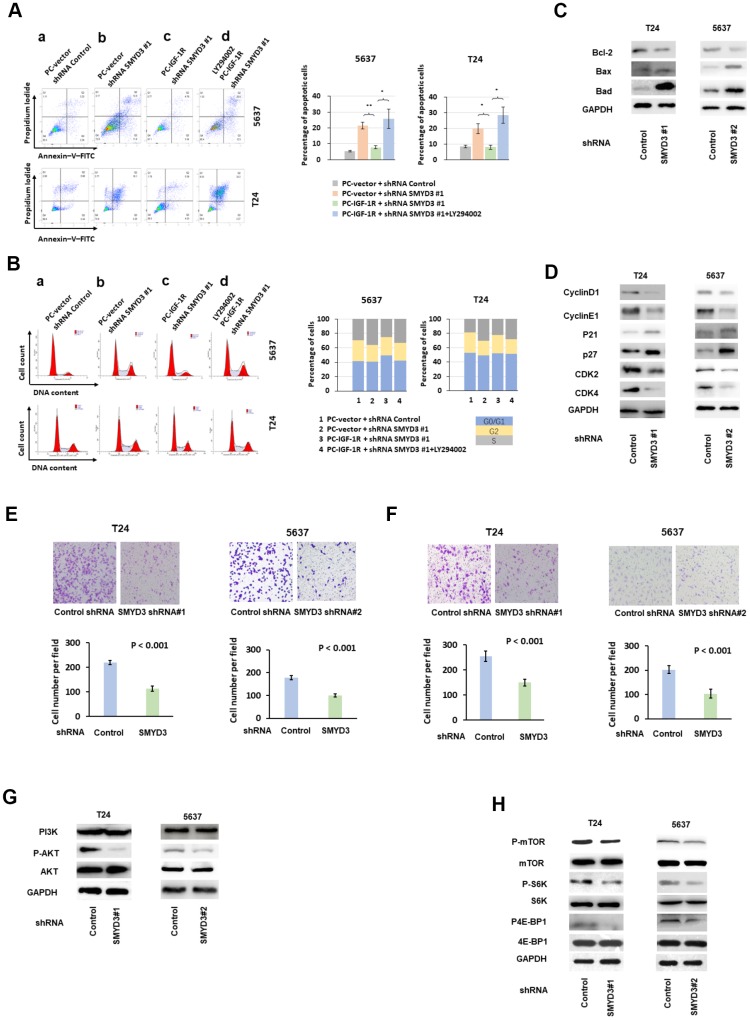
**SMYD3 promotes tumorigenic phenotypes and activates AKT/mTOR signaling pathway in BC cells.** (**A**) Left: representative images of PI/Annexin V staining of T24 and 5637 cells transfected with indicated vectors or treated with PI3K/AKT inhibitor LY294002(20μmol/L). Four independent experiments were performed for each cell line. Right: quantification of cells in apoptosis. Bar: SD, **: P< 0.01, *: P< 0.05, *t* test. (**B**) Representative examples of propidium iodide staining of T24 and 5637 cells as indicated above. Four independent experiments were performed for each cell line. The percentage of cells in each transfected population in each cycle phase was calculated (right panels). (**C**) Western blot analysis of Bcl-2, Bax and Bad protein expression in T24 and 5637 cells transfected with SMYD3-shRNA or con-shRNA. (**D**) Western blot analysis of cyclin D1, cyclin E1, p21, p27 CDK2 and CDK4 protein expression in T24 and 5637 cells transfected with SMYD3-shRNA or control shRNA. GAPDH served as a loading control. (**E**) Transwell migration assays of T24 and 5637 cell lines. Upper: representative images of Transwell migration assays of BC cells 48 h after incubation. Lower: The cells that migrated to the lower compartment were counted in by light microscopy at X 40 magnification. Tweleve representative fields were analyzed for each well after 48 h of incubation (n=4). Bar: SD, t test. (**F**) Upper: Representative images of Transwell invasion assays of BC cells 48 h after incubation. Lower: Transwell invasion assays of T24 and 5637 cell lines. The cells were counted in 12 representative fields for each well after 48 h of incubation (n=4). Bar: SD, t test. (**G**) Western blot analysis of PI3K, phosphorylated-AKT (P-AKT) and AKT protein expression in T24 and 5637 cells transfected with SMYD3 shRNA or control shRNA. Three independent experiments were performed. (**H**) Western blot analysis of p-mTOR, mTOR, p70, S6K, p4E-BP1 and 4E-BP1 protein expression in T24 and 5637 cells transfected with SMYD3 shRNA or control shRNA. GAPDH served as a loading control. p-S6K, phosphorylated S6K; p4E-BP1, phosphorylated 4E-BP1. Three independent experiments were performed.

We next asked how SMYD3 depletion inhibited tumor growth of BC cell lines. For this purpose, we examined apoptosis and cell cycle using anti-Annexin-V-FITC and propidium iodide (PI) staining in SMYD3-depleted BC cells. FACS analysis showed that depletion of SMYD3 by shRNA induced apoptosis in T24 and 5637 cells (Figure 3A, a and b). In addition, PI staining revealed that SMYD3 knockdown led to a remarkable accumulation of cells in the S phase (Figure 3B, a and b).

To elucidate the mechanism underlying the SMYD3-induced anti-apoptotic effects, we examined the impact of SMYD3 inhibition on Bcl-2, Bad and Bax expression. In BC cells treated with SMYD3 shRNA/control shRNA, we found that SMYD3 exerted its anti-apoptotic effect by up-regulating the expression of Bcl-2 and down-regulating the expression of Bax and Bad (Figure 3C). We further analyzed cell cycle regulators in BC cells and observed that SMYD3 depletion led to decreased cyclin D1, CDK4, cyclin E1, and CDK2 protein expression, but enhanced p21 and p27 expression (Figure 3D).

### SMYD3 drives migration and invasion of BC cells

Epigenetic changes of cancer cells are associated with cellular movement [17]. We thus asked whether SMYD3 played a role in the migration and invasion of T24 and 5637 cells. We determined the impact of SMYD3 inhibition on serum-induced movement using Transwell cell migration assays and found that cells stably transfected with SMYD3 shRNA exhibited reduced ability to pass through the pore (cell migration at 48 h: T24-SMYD3-shRNA#1 vs T24-Con-shRNA = 113.9 (cells per field of light microscopy at x 40 magnification) vs 219.2, *P*< 0.001, *t*-test; 5637-SMYD3-shRNA#2 vs 5637-Con-shRNA = 101.9 vs 178.3, *P*< 0.001, *t*-test) (Figure 3E). These Similar results were obtained from the cell invasion assays (cell invasion at 48 h: T24-SMYD3-shRNA#1 vs T24-Con-shRNA = 150.0 vs 255.4, *P*< 0.001, *t*-test; 5637-SMYD3-shRNA#2 vs 5637-Con-shRNA = 103.1 vs 202.2, *P*< 0.001, *t*-test) (Figure 3F).

### SMYD3 activates the AKT/mTOR signaling pathway in BC cells

We then sought to explore the molecular mechanism underlying the SMYD3-mediated pro-tumorigenic effects on BC cells. The AKT-mTOR pathway was repressed by SMYD3 depletion in T24 and 5637 cells. As shown in Figure 3G, SMYD3 inhibition by shRNA suppressed AKT phosphorylation (P-AKT), but the abundance of total AKT and PI3K protein was unchanged. Evidently, SMYD3 induced AKT activity via a post-translational modification.

We next determined a potential link between SMYD3 expression and the signaling cascade of mTOR, which is a main downstream substrate of AKT in tumorigenesis. As shown in Figure 3G, SMYD3 depletion substantially reduced the phosphorylated mTOR level in BC cells, without affecting the translation of mTOR protein. The major function of mTOR in oncogenesis is to control protein synthesis by directly phosphorylating the translational regulators eukaryotic translation initiation factor 4E -binding protein 1 (4E-BP1) and S6 kinase 1 (S6K1) [18]. SMYD3 inhibition suppressed the phosphorylation of 4E-BP1 and S6K1, but did not affect the abundance of the total 4E-BP1 or S6K1 protein in T24 or 5637 cells (Figure 3H).

### SMYD3 induces IGF-1R transcription and expression in human BC cells

**Figure 4:**
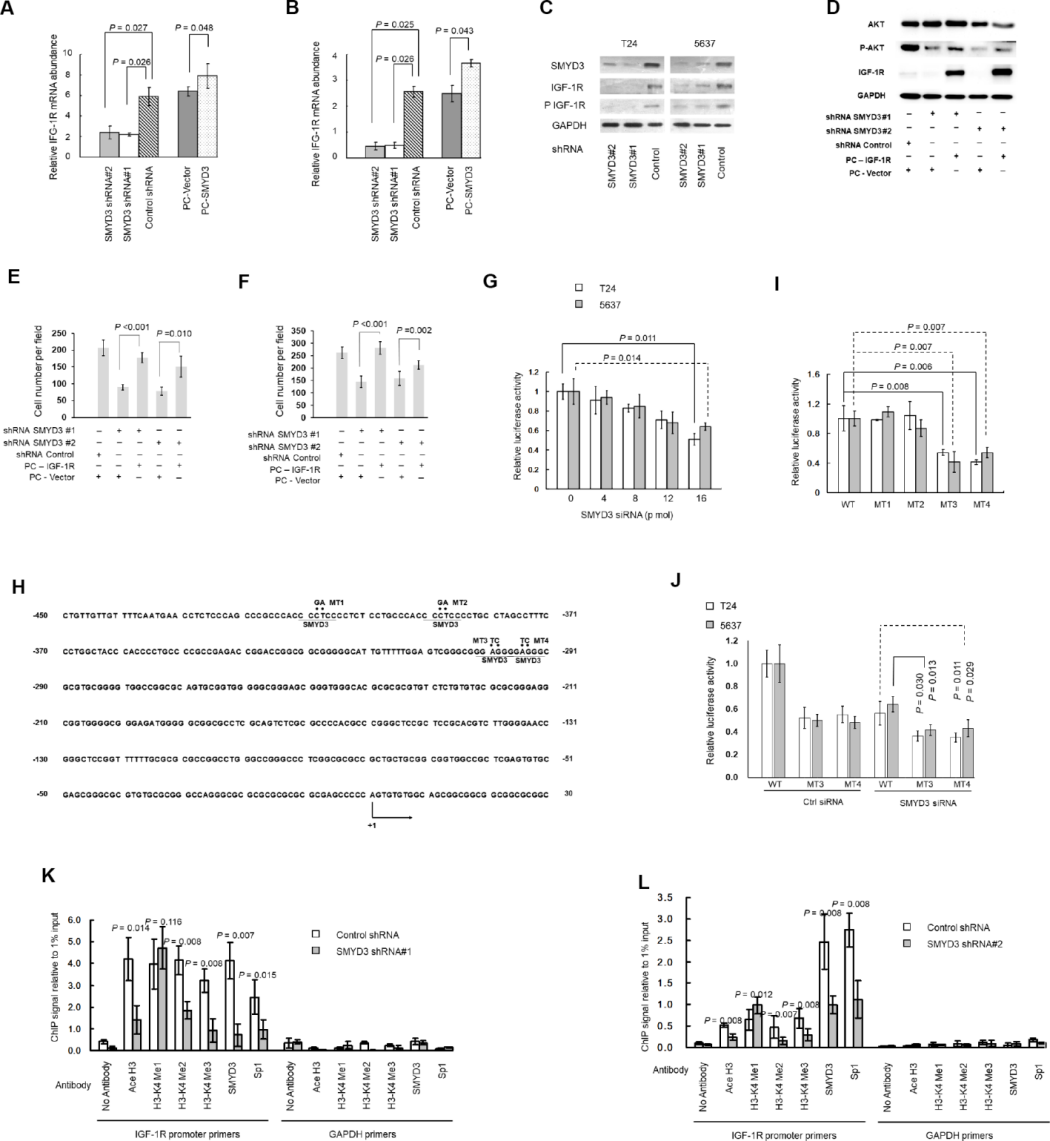
**SMYD3 induces IGF-1R transcription through promoter chromatin remodeling.** (**A**, **B**) RT-PCR of IGF-1R transcription in T24 (**A**) and 5637 (**B**) cells transfected with SMYD3 shRNA/ control shRNA or PC-vector/PC-SMYD3. The data were normalized to the mRNA abundance of β-actin. Error bars correspond to standard deviations. Wilcoxon signed-rank tests for the paired samples were used to calculate the two-sided P values based on six independent experiments. (**C**) Western blot analysis of SMYD3, IGF-1R, and phosphorylated IGF-1R (P-IGF-1R) expression in BC cells, the same transfections and blot (GAPDH and SMYD3) as Figure 2C. (n=3). (**D**) Western blot analysis of AKT, P-AKT and IGF-1R protein expression in T24 cells 48 h post transfected with indicated vectors. (**E**) Transwell migration assays of T24 cells 48 h post transfected with indicated vectors, performed as Figure 3E. (**F**) Transwell invasion assays of T24 cells 48 h post transfected with indicated vectors, performed as Figure 3F. (**G**) Increasing doses of SMYD3 siRNA were co-transfected with wild-type (WT) pGL3-IGF-1R-LUC plasmid and pRL-TK into T24 or 5637 cells. A luciferase activity assay was performed 48 h after transfection. Six independent transfections were performed. Error bars correspond to standard deviations. A Wilcoxon signed-rank test for the paired samples was used to calculate the two-sided *P* value. (**H**) Sequence of the IGF-1R core promoter region. Four potential SMYD3 binding sites are underlined. The sequence that was mutated in the transcriptional activity analysis of *cis*-acting elements (MT1–MT4) is indicated by dots, and substitutions are given above. The first nucleotide upstream of the transcription start site is indicated by +1; the arrow indicates the first nucleotide of the first exon. (**I**) WT or SMYD3 motif mutant (MT1–MT4) IGF-1R promoter activity in T24 and 5637 cells. Six independent transfections were performed. Error bars correspond to standard deviations. Wilcoxon signed-rank tests were used to calculate the two-sided P values. (**J**) SMYD3 siRNA was co-transfected with WT or mutant reporter plasmid (MT3 or MT4) into BC cells. Luciferase activity assay was performed 48 hours after transfection. Three independent experiments were performed in duplicate. Error bars: Standard deviations. Wilcoxon signed-rank tests were used to calculate the two-sided P values. (**K**, **L**) Quantitative ChIP assay for H3-K4 tri/di/monomethylation, H3 acetylation, and Sp1 and SMYD3 occupancy at the IGF-1Rpromoter in T24 (**K**) and 5637 (**L**) cells expressing SMYD3 shRNA or control shRNA. Omission of antibodies (No Antibody) was included throughout the entire experimental procedure, in addition to PCR amplification of the unrelated GAPDH gene, as an appropriate control. The data shown are from three independent experiments in triplicate. Mean values of ChIP signals are normalized to 1% input. Input control was from non-immunoprecipitated total chromatin DNA. Error bars correspond to standard deviations. Wilcoxon signed-rank tests were performed to calculate the two-sided P values. Ace H3, acetylated Histone 3; H3-K4 Me1, monomethylated H3-K4; H3-K4 Me2, dimethylated H3-K4; H3-K4 Me3, trimethylated H3-K4.

We then investigate the molecular mechanism underlying SMYD3-induced AKT activation. IGF-1R is known to be over-expressed in BC and to promote BC progression through the AKT/mTOR pathway [19]. As shown in Figure 4A and B, SMYD3 depletion reduced IGF-1R mRNA expression in BC cells, whereas ectopic expression of SMYD3 in T24 and 5637 cells slightly enhanced IGF-1R mRNA expression 48 h post-transfection (Figure 4A and 4B). Furthermore, SMYD3 knockdown abolished IGF-1R and phosphorylated IGF-1R expression (Figure 4C). To probe whether the reduced AKT activation in SMYD3-depleted cells was due to the SMYD3 shRNA-mediated down-regulation of IGF-1R, we ectopically expressed IGF-1R in T24-SMYD3-shRNA. As shown in Figure 4D, the abolished AKT phosphorylation resulting from SMYD3 depletion was clearly rescued by ectopic IGF-1R expression. To confirm SMYD3 as an oncogenic driver through activation of AKT in BC cells, we evaluated the apoptosis and cell cycle after treating IGF-1R rescued 5637-SMYD3-shRNA and T24-SMYD3-shRNA cells with a PI3K/AKT inhibitor LY294002. As anticipated, the pro-tumorigenic effects of IGF-1R rescue were reversed in FACS analysis (Figure 3A and 3B, c and d) In addition, IGF-1R over-expression also restored the SMYD3 shRNA-induced cell migration (Figure 4E) and invasion (Figure 4F) in BC cells.

To determine whether SMYD3 up-regulates IGF-1R expression at the transcriptional level, we examined the effect of SMYD3 depletion on IGF-1R promoter activity. Using a dual luciferase reporter assay system, we found that SMYD3 inhibition repressed IGF-1R promoter activity by 49.0% and 36.0% in T24 and 5637 cells, respectively (Figure 4G). SMYD3 is known to bind to a putative motif 5′-CCCTCC-3′ or 5′-GGAGGG-3′in its target promoters, and consistently, we identified four potential SMYD3 binding motifs within the IGF-1R core promoter region (-416 - +23) (Figure 4H) [20]. By mutating each SMYD3 binding site (MT1–MT4), we found that two of them (MT3 and MT4) were required for the transcriptional activity of the *IGF-1R* gene and disruption of them resulted in a significant reduction of the promoter activity in both T24 and 5637 cells (Figure 4I). In contrast, mutation of the remaining two motifs (MT1 and MT2) did not affect the basic activity of the IGF-1R promoter, indicating that they were nonfunctional. We then co-transfected SMYD3 siRNA with wide type or MT3/4 IGF-1R reporter vectors. As shown in Figure 4J, SMYD3 knockdown also inhibited the activity of MT3/4 reporter but at a less level compared with cells co-transfected with control siRNA, which further indicates that these two motifs are of functional importance for SMYD3 regulation of IGF-1R expression.

### SMYD3 inhibition leads to altered chromatin remodeling and defects in Sp1 binding at the *IGF-1R* promoter

We then determined whether SMYD3 induces IGF-1R expression by affecting chromatin remodeling and subsequent *trans*-activation. Using ChIP assay, we found that SMYD3 depletion led to decreased H3-K4 di/trimethylation coupled with slightly increased monomethylation at the *IGF-1R* promoter in both T24 and 5637 cells (Figure 4K and 4L).

Sp1 is a well-defined transcription factor of IGF-1R [20]. Because H3-K4 di/trimethylation alters chromatin folding that provides specific binding sites for certain proteins, including histone acetyltransferases, and in turn contributes to increased DNA accessibility to transcription factors, we wanted to determine whether the abolished H3-K4 hyper-methylation affected the occupancy by Sp1, a key activator for IGF-1R transcription, on IGF-1R promoter [21]. The ChIP assay showed that both H3 acetylation and Sp1 occupation was abolished when SMYD3 expression was repressed by shRNA in T24 and 5637 cells (Figure 4K and 4L). Taken together, SMYD3-mediated H3-K4 di/trimethylation is a critical event for the recruitment of histone acetyltransferase and Sp1 to the IGF-1R promoter in BC cells.

### SMYD3 forms a positive feedback loop with an IGF-1R/AKT/E2F-1 axis

**Figure 5:**
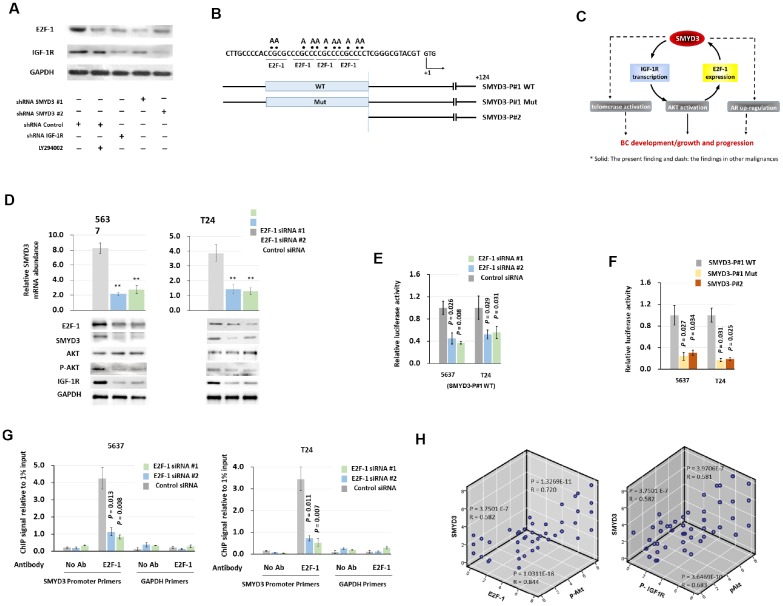
**SMYD3 forms a positive feedback loop with IGF-1R/AKT/E2F-1 axis.** (**A**) Western blot analysis of E2F-1 and IGF-1R protein expression in T24 cells 48 h post transfected with indicated vectors and treated with LY294002 (20μmol/L). (**B**) Schematic presentation of putative E2F-1–binding domains in the 5' flanking region of SMYD3. Reporter vector contained either wild type (SMYD3-P#1 WT) or mutant (SMYD3-P#1mt) E2F-1–binding sequences. +1: transcription start site. (**C**) A model of a positive feedback loop, involving SMYD3, IGF-1R and E2F-1, that sustains AKT activation, promotes BC development and progression. (**D**) qPCR and Western blot analysis of SMYD3 expression in 5637 and T24 cells transfected with E2F-1 siRNAs or control siRNA. Bars: SD. Wilcoxon signed-rank tests for the paired samples were used to calculate the two-sided *P* values based on six independent transfections. ** P < 0.01. (**E**) E2F-1 siRNA were co-transfected with WT SMYD3-P#1 into 5637 and T24 cells. Luciferase activity assay was performed 48 hours after transfection. Six independent transfections were performed. Bar: SD. A Wilcoxon signed-rank test for the paired samples was used to calculate the two-sided *P* value. (**F**) WT, E2F-1 motif mutant, or truncated SMYD3 promoter activity in 5637 or T24 cells. Three independent experiments were performed in triplicates. Bar: SD. A Wilcoxon signed-rank test for the paired samples was used to calculate the two-sided *P* value. (**G**) Quantitative chromatin immunoprecipitation (ChIP) assay forE2F-1 occupancy at the SMYD3 promoter in 5637(left) or T24 (right) cells treated with the E2F-1 siRNA or control siRNA). Omission of antibodies (No Ab) was included in the whole experimental procedure, together with the PCR amplification of unrelated GAPDH gene, as appropriate controls. Data shown are from four independent transfections. Mean values of ChIP signals are normalized to 1% input. Input control was from non-immunoprecipitated total chromatin DNA. Bar: SD. Mann–Whitney U tests were used to calculate the two-sided *P* values. (**H**) The correlation among concurrent immunostaining scores of SMYD3, E2F-1, p-IGF-1R and p-AKT in BC tissues. The Spearman test was used in analyzing the correlation.

Our results showed that SMYD3 is involved in the phosphorylation of AKT and the regulation of cycle cell checkpoint genes, however, the downstream pathway that links AKT to cell cycle transition remains elusive. Since the E2F-1 is a well-established target of this pathway and rapamycin modulates the transcriptional activity of E2F [22–25], we sought to determine the expression of E2F-1 with altered SMYD3/ IGF-1R/AKT axis. As shown in Figure 5A, both SMYD3 and IGF-1R inhibition could lead to diminished E2F-1 expression in 5637 cells; moreover, PI3K/AKT inhibitor LY294002 repressed E2F-1 expression. Our results suggest that E2F-1 is a downstream factor of the SMYD3/ IGF-1R/AKT axis.

Since there exist potential E2F-1 binding motifs in the SMYD3 promoter region (Figure 5B) and SMYD3 was a direct downstream target to RB-E2F signal- transduction pathway [26], we postulate the existence of a positive feedback loop promoting BC progression (Figure 5C). As shown in Figure 5D, E2F-1 depletion reduced SMYD3 mRNA and protein expression in BC cells. (SMYD3 mRNA abundance: 5637 cells: 26.2% (E2F-1 shRNA #1) and 32.9% (E2F-1 shRNA #2) of levels in control shRNA); T24 cells: 36.8% (E2F-1 shRNA #1) and 33.5% (E2F-1 shRNA #2) of levels in control shRNA). In addition, E2F-1 depletion reduced the abundance of phosphorylated AKT and IGF-1R in BC cells, further supporting the existence of the positive feedback loop (Figure 5D).

To delineate the molecular mechanism causing transactivation of SMYD3, we constructed reporter vectors containing wild-type binding elements (SMYD3-P#1 WT), reporter plasmids without the binding elements (SMYD3-P#2) and mutant reporter plasmids containing substitutions in all four binding elements (SMYD3-P#1Mut) as described by Tsuqe M. et al. [26] (Figure 5B). As shown in Figure 5E, E2F-1 inhibition using shRNA led to a significant decrease in the promoter activity (5637 cells: 45.1% (SMYD3 shRNA-1) and 37.0% (SMYD3 shRNA-2) of levels in scrambled control; T24 cells: 52.0% (SMYD3 shRNA-1) and 56.2% (SMYD3 shRNA-2) of levels in scrambled control), respectively. The reporter activity of SMYD3-P#2 was 29% of SMYD3-P#1, and that of SMYD3-P#1Mut was 24.1% of SMYD3-P#1 in 5637 cells; and 19.0% and 17.1% in T24 cells, respectively (Figure 5F), indicating that this motif is a target of E2F-1. ChIP assays were then employed to validate whether E2F1 could interact with the promoter of SMYD3. As expected. In accordance with the SMYD3 reporter assay results above, E2F1 was significantly enriched at the SMYD3 promoter region in 5637 and T 24 cells (Figure 5G). Finally, we investigated whether p-AKT, p-IGF-R and E2F1 levels correlated with SMYD3 levels in the BC patient samples and IHC showed positive correlations (Figure 5H).

## DISCUSSION

In the present study, we show that SMYD3 as a poor prognostic indicator is widely over-expressed in BC tumors and plays a pro-tumorigenic role in BC pathogenesis. More importantly, SMYD3 activates the AKT signaling through inducing IGF-1R expression; while AKT stimulated SMYD3 expression in an E2F-1-dependent manner, thus forming a positive feedback circuit leading to the hyperactivated AKT signaling in BC cells. This positive feedback loop is required to promote proliferation, survival, migration and invasion of BC cells. Thus, disrupting this loop may offer an effective strategy for BC therapeutic intervention.

IGF-1R, a receptor tyrosine kinase, is now considered a potential cellular oncogene that plays a key role in various cellular processes, such as proliferation, survival, and transformation as well as cell invasion and migration [27, 28]. It has now become a very attractive target for cancer therapy, and several compounds targeting IGF-IR are currently in phase III clinical trials [29, 30]. IGF-1R expression is undetectable or very weak in normal bladder tissues and over-expressed in BC [8]. It has long been known that IGF-1R is critical for BC progression and promotes BC growth through AKT activation, however, the underlying mechanism of IGF-1R deregulation is elusive [19, 31]. Several studies have shown that epigenetic aberrations play important roles in the pathogenesis of BC, but few of them have addressed the epigenetic regulation of IGF-1R expression [32]. It has been proposed that H3-K4 di-/trimethylation could serve as a specific binding site of proteins or protein complexes and was a critical event that recruits histone acetyltransferases (HAT) and promotes transcription factor/s access to the promoter of target genes [33]. The proximal promoter of IGF-1R gene harbors GC-boxes, where transcription factor Sp1 plays a key role in regulating IGF-1R transcription [34]. Here we found diminished histone H3 acetylation and impaired occupancy of Sp1 on the IGF-1R promoter following SMYD3 silencing and subsequent inhibition of H3-K4 di-/trimethylation in the examined BC cells. To the best of our knowledge, this is the first study showing that the histone methyltransferase SMYD3 is involved in the regulation of IGF-1R transcription, through which an oncogenic-effect is exerted in BC.

SMYD3 is aberrantly over-expressed in the majority of cancers and has been shown to exert oncogenic effects through various mechanisms. First, SMYD3 binds to its motif/s located in the promoter region of the target genes and induces transcription via its H3-K4 methylation activity. SMYD3 may also form a complex with RNA polymerase II and transactivate target genes [35, 36]. For instance, SMYD3 directly targets the androgen receptor and telomerase gene, thereby promoting development and progression of prostate cancer and other human malignancies [15, 37]. In addition, as a H3-K4 methyltransferase, SMYD3 trans-activates many genes, including MMP-9, Bcl-xL, WNT10B, and Nkx2.8, all of which play critical roles in cancer development and/or progression [14]. Second, SMYD3-mediated methylation of MAP3K2 at lysine 260 potentiates activation of the Ras/Raf/MEK/ERK signaling module and promotes the formation of Ras-driven carcinomas [13]. Third, SMYD3 directly interacts with the ligand binding domain of estrogen receptor (ER) and functions as a coactivator of ER alpha and potentiates ERalpha activity [38]. Here AKT activation contributes to the pro-tumorigenic activity mediated by SMYD3 in BC, which reveals a novel oncogenic effect of SMYD3. Androgen receptor (AR) signaling is known to be involved in the etiology and progression of BC [39], while the AR gene is a direct target of SMYD3 in prostate cancer. Therefore, it is of interest to determine if this is the case in BC.

We also noticed both cytoplasmic and nuclear SMYD3 distribution in primary BC cells, which raises a number of questions. What does cytoplasmic SMYD3 do? Does it methylate non-histone proteins? More importantly, does cytoplasmic SMYD3 contribute to its oncogenic activity in BC [40]? In the study presented here, we observed a remarkable increase in SMYD3 expression in BC tissues. Consistent with our findings, endogenous expression of SMYD3 was also reported to be significantly up-regulated in a majority of colorectal carcinoma, hepatocellular carcinoma, prostate cancer, and breast cancer specimens [15, 26, 41]. Little is known about the mechanism(s) underlying SMYD3 over-expression in those malignancies. E2F-1 was shown to be involved in the transcriptional activation of the *SMYD3* gene, and a variable number of tandem repeat polymorphisms of an E2F-1 binding element in the SMYD3 promoter region was considered as a risk factor for familial breast cancers [26]. It is thus likely that the SMYD3-IGF-1R-AKT/MTOR-E2F1 axis identified in BC tumors also plays important roles in the development and progression of other malignancies. Of note, it was reported that E2F-1 directly bond and transactivated the IGF-1R promoter in immortalized prostate epithelial cells P69 and M12 [42]. In our study, we found that E2F-1 activated the IGF-1R via activation of SMYD3 in BC cells. SMYD3 is also highly expressed in prostate cancer [15], E2F-1 could exert its IGF-1R transactivation effect through both direct and indirect pathways.

Our study had some limitations. (i) The number of clinical specimens included in the present study was not large, and follow-up time was not long enough. A solid conclusion of SMYD3 as a prognostic factor for BC depends on further clinical observations at multi-hospitals. (ii) The present findings reveal the critical role of SMYD3-mediated activation of the IGF-1R-AKT axis in BC, however, based on published reports, it is likely that SMYD3 contributes to BC pathogenesis via multiple mechanisms, which calls for further studies. We previously reported that telomerase reverse transcriptase (hTERT) and AR were direct targets of SMYD3 in cancers [15, 37]. Because both hTERT and AR are involved in the pathogenesis of BC [43–45], SMYD3 could exert its pro-tumorigenesis function via these defined mechanisms (Figure 5H).

In summary, we show that SMYD3 over-expression is widespread in BC and is a poor prognostic indicator. Through a positive feedback loop with IGF-1R/AKT and E2F-1 pathway, SMYD3 exhibit strong pro-tumorigenic phenotype in BC cells. Our findings provide not only profound insights into SMYD3-mediated oncogenic activity but also present a unique avenue for treating BC by directly disrupting this positive feedback loop.

## MATERIALS AND METHODS

### Patients and tissue specimens

Sixty-five BC (urothelial carcinoma) patients undertaken radical cystectomy and seventy BC patients (urothelial carcinoma) undertaken TURBT at Shandong University Qilu Hospital were included in the study, between 2011 and 2015. The diagnosis was confirmed by histopathological examination. Clinical staging was determined according to the tumor, node, metastasis (TNM) classification of the International Union against Cancer [46]. Patients′ clinical characteristics are summarized in Table 1 and Supplementary Table 2. The study on tissue of patients and animals was approved by the ethics committee of Shandong University Qilu Hospital, and written informed consent was obtained from each participant.

### Cell lines, culture conditions and reagents.

Human BC cell lines T24 (ATCC, Manassas, VA) and 5637 (Chinese Academy of Medical Sciences Shanghai Cell Bank) were cultured at 37°C/5% CO_2_ in RPMI 1640 (Invitrogen, Carlsbad, CA) containing 10% FBS (Invitrogen), 100 U /ml penicillin (Sigma-Aldrich, St Louis, MO) and 100 μg/ ml streptomycin (Sigma-Aldrich). The PI3K inhibitor LY294002 was obtained from Millipore (MA, USA).

### siRNA treatment and shRNA transfection

Chemically modified stealth siRNA (Invitrogen) and control siRNA were purchased from Invitrogen. SMYD3 shRNA vectors (GV102, SMYD3-shRNA #1, #2 and Control shRNA) were purchased from GeneChem (Shanghai, China). Transfection of the shRNA-SMYD3 vector was performed using LipofectAMINE2000 (Invitrogen). Stable shRNA-expressing colonies were selected using G418 (Sigma-Aldrich). The sequences of siRNA and shRNA are shown in Supplementary Table 1. PC vector expressing human wild-type IGF-1R cDNA was constructed by Genechem (Shanghai, China) [47].

### RNA extraction, reverse transcription and real-time quantitative PCR (QPCR)

Total cellular RNA was extracted from tissue specimens using TRIzol reagent (Invitrogen) according to the manufacturer's instruction. A total of 1 μg of RNA was used for reverse transcription with M-MLV reverse transcriptase (Thermo Fisher Scientific, Lithuania). The PCR primers are shown in Supplementary Table 1. QPCR was performed using SYBR Green PCR Master Mix (Applied Biosystems, Foster City, CA) in a 7500 Real-Time PCR System (Applied Biosystems). Relative levels of SMYD3 mRNA in given samples were calculated using the 2−ΔΔCt method according to threshold cycle values.

### Western blots and immunohistochemisrty (IHC)

Western blots and IHC were performed as described [15]. Additional details are in the Supplementary Materials and Methods.

### Cell proliferation and xenograft tumor growth

T24 and 5637 cells stably expressing SMYD3 shRNA or control vector were plated into six-well plates (200 cells per well) and incubated for 14 days. Wells were stained with Giemsa (Sigma-Aldrich), and colonies with >50 cells were counted.

Five-week-old male BALB/c nude mice were from Shanghai Slac (China). T24 or 5637 cells stably expressing SMYD3 shRNA or control shRNA were suspended in 150 μL of PBS and inoculated subcutaneously in the inguinal area at 0.8 × 10^6^ cells per injection site. After 6 weeks, the mice were sacrificed, and tumors were removed, weighed and analyzed.

### Transwell assays

Transwell assays were performed using a Transwell chamber migration assay (8-μm pore size, Costar, New York, NY). For migration assays, T24 and 5637 cells stably expressing SMYD3 or control shRNA were trypsinized and transferred to the upper chamber in 100 *μ*l of serum-free medium containing 5 × 10^4^ cells and incubated for 24, 48 and 72 h. The lower chamber was filled with medium supplemented with 10% FBS as a chemoattractant. The membranes were fixed and stained using 0.1% crystal violet. The invading cells in 5 randomly selected high-power fields (× 400) were counted under a microscope. For invasion assays, Transwell chambers were first covered with 50 μl of Matrigel (dilution at 1 : 5; BD biosciences) and the rest of experimental procedure was the same as that for the migration assays.

### Flow cytometry (FACS) analysis of apoptosis and cell cycle

BC cells were incubated with PI and Annexin V-fluorescein isothiocyanate (BestBio, Shanghai, China) at room temperature. FACS was immediately performed according to the manufacturer's instructions.

### Luciferase reporter assay

The reporter plasmid, pGL3-IGF-1R-LUC is an IGF-1R promoter construct containing a short fragment of the IGF-1R promoter from -416 to +23 base-pairs, subcloned upstream of firefly luciferase cDNA, as described previously [20]. Cells cultured in 24-well plates were transfected with the IGF-1R reporter construct or mutant variants and siRNA. A renilla luciferase-containing plasmid, pRL-TK, which is driven by a thymidine kinase promoter, was always included in the transfection to control for transfection efficiency. The luciferase activity was determined using a dual luciferase reporter assay system following the manufacturer’s instructions (Promega, WI, USA).

### Chromatin immunoprecipitation (ChIP)

ChIP was performed using a ChIP Assay Kit (Millipore) according to the manufacturer’s instructions. Briefly, cells were cross-linked by incubation with 1% (v/v) formaldehyde-containing medium for 10 min at 37°C and then sonicated to obtain soluble chromatin with DNA fragments between 400 and 1,000 base-pairs. Antibodies against mono/di/trimethylated H3-K4 (Millipore), acetylated histone H3 (Millipore), SMYD3 (Santa Cruz Biotechnology), and Sp1 (Santa Cruz Biotechnology) were used to precipitate DNA fragments bound by their corresponding elements. The protein-DNA complex was precipitated with protein A sepharose beads (Millipore), eluted, and reverse cross-linked. Following a treatment with protease K (Sigma-Aldrich), the samples were extracted with phenol-chloroform and precipitated with ethanol. The recovered DNA was re-suspended in TE buffer and used for QPCR amplification with primer sets (Supplementary Table 1). GAPDH was used as a negative control.

### The cancer genome atlas (TCGA) dataset

The TCGA Research Network is available at http://cancergenome.nih.gov/. The datasets for BC cases within the TCGA database were downloaded at Memorial Sloan Kettering Cancer Center cBioPortal for cancer genomics as of May 5, 2019 [48].

### Statistical analysis

Non-parametric data between paired samples, including QPCR, quantitative ChIP, and Transwell assays, were analyzed using a Wilcoxon signed-rank test. Parametric data between paired groups were analyzed using a *t*-test. Associations between SMYD3 immunostaining level and clinicopathological parameters were analyzed with a *χ*^2^-test (IBM SPSS Statistics, version 23). The Spearman rank correlation coefficient was used as a statistical measure of association. The Kaplan–Meier method with the log-rank test was applied for overall survival (OS) and progression-free survival (PFS). Cox regression analysis was performed to evaluate the survival data. All statistical tests were two-sided. A *P* value <0.05 was considered statistically significant. We used a Bonferroni correction to adjust for multiple tests and only considered values less than 0.05/n (n: the number of hypotheses in a test) to be statistically significant.

## Supplementary Material

Supplementary Methods

Supplementary Tables
