# Supplementary material for: High expression of SMYD3 indicates poor survival outcome and promotes tumour progression through an IGF-1R/AKT/E2F-1 positive feedback loop in bladder cancer
Source: Aging (Albany NY). 2020 Feb 1;12(3):2030–48. doi: 10.18632/aging.102718 (PMC7041758; doi:10.18632/aging.102718)
Supplement: Supplementary Methods [file aging-12-102718-s001..pdf]

SUPPLEMENTARY METHODS

Western blots

Total cellular protein was extracted with lysis buffer (DBI Bioscience, Shanghai, China). Protein concentration was quantified using a BCA Protein Quantitative Kit (Beyotime, China). Briefly, 30 µg of protein was resolved by SDS–PAGE, and transferred to a PVDF membrane (Millipore, Billerica, MA). The membrane was blocked with 5% skim milk and then probed with primary antibodies against SMYD3 (ab 82577, Abcam), PI3K (YP0224, Immunoway, DE,USA), AKT (9271S, Cell Signaling Technology, CST, MA), p-AKT (9275S, CST), p21 (2947P, CST), p27 (3686P, CST), CDK2 (2546P, CST), CDK4 (12790, CST), cyclin D1 (2926S, CST), cyclin E1 (4129S, CST), Bcl-2 (2876S, CST), Bax (5023P, CST), mTOR (2983, CST), p-mTOR (5536, CST), p-p70 (9234, CST), 4EBP1 (9452S, CST), p4EBP1 (2855, CST), E2F-1 (3742, CST), IGF-1R (C-20, Santa Cruz Biotechnology, Santa Cruz, CA, USA), p-IGF-1R (2B9, Santa Cruz), GAPDH (Santa Cruz). The membranes were incubated with anti-mouse or anti-rabbit IgG (Dako, Denmark) and developed with an ECL Kit (WBKLS0100, Millipore, Billerica, MA).

IHC

5µm paraffin sections were prepared for IHC staining. Deparaffinization was performed with xylene, tissue sections were rehydrated, and then endogenous peroxidase activity was quenched using 3% hydrogen peroxide in methanol. Antigen retrieval was performed by boiling in a microwave oven in 10 M citrate buffer (pH 6.0). Blocking non-specific binding was achieved using 5% bovine serum albumin. The slides were incubated with anti-SMYD3 antibody (ab 16027, Abcam, Cambridge, UK; 1 : 150 dilutions). After washing, the slides were incubated with horseradish peroxidase-conjugated goat anti-rabbit IgG (GK500610A, Gene Tech, Shanghai, China). Signals were developed with DAB Horseradish Peroxidase Color Development Kit (Gene Tech, Shanghai, China). The slides were scored by two independent pathologists, who were blind to the patient data, according to the intensity of SMYD3 staining. The intensity of immunostaining was scored on a scale from 0 (no staining), 1+ (week staining), 2+ (moderate staining) to 3+ (strongest staining), respectively.
