# Supplementary material for: High expression of SMYD3 indicates poor survival outcome and promotes tumour progression through an IGF-1R/AKT/E2F-1 positive feedback loop in bladder cancer
Source: Aging (Albany NY). 2020 Feb 1;12(3):2030–48. doi: 10.18632/aging.102718 (PMC7041758; doi:10.18632/aging.102718)
Supplement: Supplementary Tables [file aging-12-102718-s002..pdf]

SUPPLEMENTARY TABLES

Supplementary Table 1. RT-PCR, ChIP primer sets and siRNA/shRNA sequences used in this study.

Primer sequences for RT-PCR

IGF-1R

5′ –ACC CGG AGT ACT TCA GCG CT - 3′ (forward)

5′ –CAC AGA AGC TTC GTT GAG AA - 3′ (reverse) .

GAPDH

5′ - AAAGGGCCCTGACAACTCTT - 3′ (forward)

5′ - GGTGGTCCAGGGGTCTTACT - 3′ (reverse) .

SMYD3

5′-CCATATGCTCACTCAGATGATGT-3′ (forward)

5′-GTGTATCATCTCCACAGAGAGTT-3′ (reverse)

β-actin

5′-AGTTGCGTTACACCCTTTCTTG-3′ (forward)

5′-CAC CTTCACCGTTCCAGTTTT-3′ (reverse)

Primer sequences for ChIP assay

GAPDH

5′ - AAA GGG CCC TGA CAA CTC TT - 3′ (forward)

5′ - GGT GGT CCA GGG GTC TTA CT - 3′ (reverse) .

IGF-1R

5′ –TAG CCT TTC CCT GGC TAC CCA- 3′ (forward)

5′ –GGT TCC CCA AGA CGT GCG GA- 3′ (reverse)

siRNA sequences

E2F-1 siRNA 1 5′-AUG CUA CGA AGG UCC UGA CAC GUC A-3′

E2F-1 siRNA 2 5′-AAA GUU CUC CGA AGA GUC CAC GGC U-3′

Scramble siRNA: 5' - CCU ACA UCC CGA UCG AUG AUG UUG A - 3'

SMYD3 shRNA targets

shRNA #1 5′ - AGC CTG ATT GAA GAT TTG ATT - 3'

shRNA #2 5′ - GCT TCC CGA TAT CAA CAT CTA - 3'

Control: 5' - CCU ACA UCC CGA UCG AUG AUG UUG A - 3'

Supplementary Table 2. SMYD3 expression in relation to clinicopathologic variables in 70 BC patients undertaken TURBT.

Variable	Total	SMYD3 staining (%)	*P*-value	
	0 – 1	2 – 3		
Sex				
Male	47 (67.1)	40 (57.1)	7 (10.0)	1.000
Female	23 (32.9)	19 (27.1)	4 (5.7)	
Age, years (median 67)				
< 67	39 (55.7)	34 (48.6)	5 (7.1)	0.456
≥67	31 (44.3)	25 (35.7)	6 (8.6)	
Muscle invasive				
No	46 (65.7)	43 (61.4)	3 (4.3)	0. 010
Yes	24 (34.3)	16 (22.9)	8 (11.4)	
Histological grade				
G1	29 (41.4)	28 (40.0)	1 (1.4)	
G2	25 (35.7)	21 (30.0)	4 (5.7)	0.006
G3	16 (22.9)	10 (14.3)	6 (8.6)	（G1/2vsG3）

0–1: low-SMYD3 expression; 2–3: high-SMYD3expression. Associations between SMYD3 immunostaining level and clinicopath-oncogical parameters were analyzed with χ^2-test.
